# Pediatric Risk of Mortality III Score in Predicting Mortality Among Diabetic Ketoacidosis Patients in a Pediatric Intensive Care Unit

**DOI:** 10.7759/cureus.19734

**Published:** 2021-11-19

**Authors:** Sadam H Baloch, Prof Mohsina N Ibrahim, Pooja D Lohano, Murtaza A Gowa, Shazia Mahar, Roshia Memon

**Affiliations:** 1 Paediatrics and Endocrinology, National Institute of Child Health Karachi, Karachi, PAK; 2 Paediatric Critical Care, National Institute of Child Health Karachi, Karachi, PAK

**Keywords:** pediatric risk of mortality (prism) iii score, prism score, pediatric intensive care unit (picu), diabetic keto acidosis, type i diabetes mellitus

## Abstract

Background

Diabetic ketoacidosis (DKA) is one of the most common complications of type 1 diabetes. Mortality is not uncommon in DKA, mostly in younger children with severe DKA and those complicated with cerebral edema. Early identification of high-risk patients can help in timely interventions to improve the outcome of DKA. Pediatric Risk of Mortality (PRISM III) is a standard scoring system to objectively predict the prognosis and outcome of pediatric intensive care unit (PICU) patients.

Objective

To predict the need for inotrope and mechanical ventilation and mortality rate using PRISM III in DKA patients admitted to PICU.

Methods

A prospective observational study was conducted in the PICU of the National Institute of Child Health, Karachi, from February 2020 to September 2021 involving 114 children. PRISM III scoring protocol was applied. A PRISM III score of >8 predicted higher mortality risk.

Results

The mean PRISM III score was 6.56 ± 3.18 with 30 (26.3%) children having a score >8. Of the 30 (26.31%) patients with >8 PRISM III scores, 14 (46.67%) needed inotropic support, 6 (20%) needed mechanical ventilation, and there were eight (26.67%) mortalities. There was no reported mortality among patients with a PRISM III score ≤8. All differences were statistically significant (p < .05).

Conclusion

PRISM III is a highly sophisticated scoring system that can aid clinicians in the early prediction of adverse clinical outcomes in patients with DKA. Robust scientific evidence supporting its clinical application can help practically improve the outcome of DKA in young patients.

## Introduction

International Diabetes Federation estimates that, globally, 98,200 children of ages 15 years and younger develop type 1 diabetes mellitus (T1D) every year [1]. Diabetic ketoacidosis (DKA) is one of the most common as well as the most serious and life-threatening complications of T1D. The incidence of DKA as reported in a systematic literature review is 0-56 per 1000 person-years [2]. DKA is characterized by hyperglycemia (blood sugar levels >200 mg/dL or 11.1 mmol/L), metabolic acidosis (venous pH <7.3, and/or serum bicarbonate <15 mEq/L) with associated glycosuria, ketonuria, and ketonemia [3]. Overall pediatric mortality in DKA ranges from 0.15% to 0.35% in developed countries such as Canada, the United States, and the United Kingdom and from 3.4% to 13.4% in developing countries such as India, Pakistan, and Bangladesh [4-7]. Mortality is not uncommon in DKA. Younger children with severe DKA and those complicated with cerebral edema are more susceptible to mortality risks of DKA [8]. In developed countries, about 5.5% of cases complicate into cerebral edema while in developing countries the occurrence is reported to be as high as 24-26% [9]. Other causes of death in DKA include acute renal failure, sepsis, and shock.

With DKA and other illnesses requiring critical care, the protocols and practice guidelines are still evolving in the pediatric sector. This population is vulnerable, necessitating standard care for medically and surgically ill children. Most care providers in pediatric intensive care units (PICU) rely on their past experiences, subjective measurements, and clinical judgment to decide care protocols [10]. To bridge this gap, clinicians and researchers have practically applied scoring systems to help objectively predict the prognosis and outcome of PICU patients. The physiologic stability score index was one of the first scoring systems used initially in critically ill children for the prediction of outcome. It comprised 14 variables at that time. Later in 1996, it was altered to Pediatric Risk of Mortality (PRISM III) with the addition of three more variables. It is a widely accepted, standardized scoring system against which other scoring systems are compared. It has been validated in multiple studies, mostly in developed countries [11,12], but also in low-income settings [13,14]. While local studies have studied its performance in PICU patients recently [13], our study is unique as it took into consideration a very common subset of the PICU population, i.e. DKA patients. The objective is to predict the need for inotrope and mechanical ventilation and mortality rate using PRISM III in DKA patients admitted to PICU.

## Materials and methods

A prospective observational study was conducted in the PICU of the National Institute of Child Health (NICH). It is the largest public, pediatric, tertiary care hospital in Karachi. The Ethical Review Committee of NICH approved the study and it was conducted from February 2020 to September 2021.

Non-probability consecutive sampling technique was utilized. The sample size was calculated using the WHO sample size calculator. With an 8% prevalence of DKA, 5% margin of error, and 95% confidence interval, a sample of 113 was calculated. Children of age 1-15 years admitted to the PICU for management of DKA for more than 24 hours were included in this study. Exclusion criteria included children admitted for less than 24 hours, children who died within 12 hours of hospital admission, children who have had a procedure of cardiopulmonary resuscitation during the hospital stay, and children whose guardians did not consent to participate in the study.

Data was collected using a semi-structured proforma. It comprised of patient demographic information including gender and age. Clinical factors included in the study were: body weight, known/unknown status of diabetes, admission through emergency or the outpatient departments, presenting complaints, duration of hospital stay, and presence of urinary ketones. The outcome was assessed through three variables: the need for inotropes, the need for mechanical ventilation, and mortality. PRISM III scoring protocol was applied for all patients. A PRISM III score of ≤8 translates into a low risk of mortality and >8 predicts a higher risk of mortality.

Data were analyzed using IBM SPSS Statistics for Windows, Version 22.0 (Released 2013; IBM Corp., Armonk, New York). After summarizing, continuous variables were presented as mean and standard deviation (SD) and compared using an independent sample test. Categorical variables were presented as frequencies and percentages and compared using the chi-square test. p ≤ .05 was considered statistically significant.

## Results

A total of 114 patients were included in this study. There were more male children (n=68) as compared to females (n=46). The average age of the study sample was 9.10 ± 3.64 years with 55 (48.24%) in the 5-10 years age group. Their demographic characteristics are summarized in Table 1.

**Table 1:** Demographic characteristics of the study sample (n=114) SD: Standard deviation

Patient demographic characteristics		Frequency (%)
Gender	Male	68 (59.65%)
	Female	46 (40.35%)
Age in years	Mean ± SD	9.10 ± 3.64
	<5 years	16 (14%)
	5-10 years	55 (48.24%)
	11-15 years	43 (37.72%)
Bodyweight in kilograms	Mean ± SD	22.99 ± 8.77

Almost all children (96.5%) were admitted through the emergency department. There were 43 (37.7%) patients who were newly diagnosed with diabetes during this hospital stay. Respiratory distress was the most frequent complain (n=92; 80.7%) followed by polyuria and polydipsia (n=66; 57.9%) each. The mean days of PICU stay in the study were 4.61 ± 2.82 with 89 (78.1%) children staying for ≤5 days. The mean PRISM III score was 6.56 ± 3.18 with 30 (26.3%) children having an adverse score (>8). There were eight (7.0%) mortalities, eight (7.0%) required mechanical ventilation, and 16 (14.0%) required inotrope support (Table 2).

**Table 2:** Clinical characteristics of the study sample (n=114) PICU: pediatric intensive care unit; PRISM III: Pediatric Risk of Mortality III

Patient Clinical Characteristics		Frequency (%)
Admission route	Emergency	110 (96.49%)
	Clinic	4 (3.51%)
Diabetes status	Known diabetes	71 (62.28%)
	Newly diagnosed	43 (37.72%)
Clinical complains	Respiratory distress	92 (80.70%)
	Polyuria	66 (57.89%)
	Polydipsia	66 (57.89%)
	Abdominal pain	56 (49.12%)
	Vomiting	45 (39.47%)
	Fever	41 (35.96%)
Duration of PICU stay in days	Mean ± SD	4.61 ± 2.82
	≤5 days	89 (78.07%)
	>5 days	25 (21.92%)
PRISM III score	Mean ± SD	6.56 ± 3.18
	≤8	84 (73.68%)
	>8	30 (26.31%)
Outcome	Need for inotrope	16 (14.03%)
	Need for mechanical ventilation	8 (7.01%)
	Mortality	8 (7.01%)

As shown in Table 3, PRISM III scores were correlated with patient outcomes. It was seen that of the 30 children with >8 PRISM III scores, 14 (46.67%) needed inotropic support, six (20%) needed mechanical ventilation, and there were eight (26.67%) mortalities. There was no reported mortality among patients with a PRISM III score ≤8. All differences were statistically significant (p < .05).

**Table 3:** Correlation of PRISM III score with patient outcome PRISM III: Pediatric Risk of Mortality III

PRISM III Score	Need for inotrope			Need for mechanical ventilation			Mortality		
	Yes	No	P value	Yes	No	P value	Yes	No	P value
≤8	2 (2.38%)	82 (97.62%)	0.000	2 (2.38%)	82 (97.62%)	0.004	0	84 (100%)	0.000
>8	14 (46.67%)	16 (53.33%)		6 (20%)	24 (80%)		8 (26.67%)	22 (73.33%)	

## Discussion

In recent times, with a drastic increase in the incidence of T1D, DKA is also more commonly encountered in hospital settings; especially as the first presentation in these children. Not only pediatric emergencies but also PICUs are seeing a higher influx of DKA patients. The results of a local retrospective review indicated that the rate of PICU admission for DKA increased from 1.8% in 2010 to 3.4% in 2015 in Pakistan [15].

Our study reported a male dominance in DKA patients, which is in contrast to the study by Abbas and colleagues, which supports female predominance with hormonal influences and lesser preference for female care [15]. However, the work of Mirza et al. reported a male predominance [6] and Nikhila et al. reported more females (52%) [9]. There were 54% males in Ongun et al. [16]. The mean age in our sample was 9.10 ± 3.64 years. In Abbas et al., the mean age was 8.1 ± 4.6 years [15], in Ongun et al. it was 11.31 ± 4.18 years [16] and in Lopes et al., it was 10.2 ± 2.9 years [17]. Most children of our sample were of age five years or more; similar results were seen in Abbas et al. [15]. The mean body weight in our sample was 22.99 ± 8.77 kg which is comparable to 25.22 ± 13.76 kg as reported in Abbas et al [15]. However, Ongun et al. reported a higher mean body weight (35.97 ± 16.31 kg) [16]. In our study, 43 (37.7%) received their diagnosis of T1D during this hospital stay. In other studies, 60-75% of DKA patients were newly diagnosed [15-17].

In our study, the mortality rate was 7%, 7% of patients needed mechanical ventilation, and 14% required inotrope support. Comparatively, in Abbas et al., 35% of patients needed inotropic support, 30% needed mechanical ventilation, and the mortality rate was 5.4% [15]. The mortality rate in Syed et al. was 3.4% [6] and 1.9% in Lopes et al. [17]. The mean PRISM III score in our sample was 6.56 ± 3.18. A high PRISM III score was seen in 26% of patients. All mortalities in our study were from patients with a high PRISM III score (>8). A high PRISM III score was statistically related to the need for inotropic support, need for mechanical ventilation, and mortality. The mean PRISM III score in Ongun et al. was 11.87 ± 5.47. Although it is higher than ours, they did not report any mortality and only one patient in their study required mechanical ventilation [16]. Nikhila et al. reported a very high mortality rate (34%) [9]. The mean PRISM III score of their deceased sample was significantly higher than the sample that was discharged (30.65 ± 7.50 vs. 12.58 ± 7.95) (p < .001) [9].

PRISM III score was observed to be a valuable and accurate predictor of adverse outcomes including mortality in DKA. Although PRISM III has been extensively utilized in predicting mortality in PICU patients irrespective of their diagnoses [5,11,13,14], there is limited data on its utilization in DKA patients [9,15,16]. PRISM III is a highly sophisticated scoring system that can aid clinicians in the early prediction of adverse clinical outcomes. Robust scientific evidence supporting its clinical application can help practically improve the outcome of DKA in young patients.

## Conclusions

DKA is commonly encountered in pediatric emergencies and intensive care units. Complications in DKA are not uncommon which worsen the overall prognosis of the illness. PRISM III has proven to be a valuable tool to objectively predict the outcome in these patients. Effective utilization of this scoring system can help in the early identification of high-risk groups, which translates into rigorous measures to improve patient outcomes.
